# Dementia Is Induced via the AGEs/Iba1/iNOS Pathway in Aged KK-Ay/Tajcl Mice

**DOI:** 10.3390/life13071540

**Published:** 2023-07-11

**Authors:** Keiichi Hiramoto, Masashi Imai, Shota Tanaka, Kazuya Ooi

**Affiliations:** Department of Pharmaceutical Sciences, Suzuka University of Medical Science, Suzuka 513-8670, Japan; hiramoto@suzuka-u.ac.jp (K.H.); dp23001@st.suzuka-u.ac.jp (M.I.); stanaka@suzuka-u.ac.jp (S.T.)

**Keywords:** type 2 diabetes, advanced glycation end products, ionized calcium binding adapter protein 1, tumor necrosis factor-α, inducible nitric oxide synthase

## Abstract

The onset and exacerbation of dementia have been observed in elderly patients with type 2 diabetes. However, the underlying mechanism remains unclear. In this study, we investigated the effects of aging on the cognitive function in a mouse model of type 2 diabetes. Pathogen-free KK-Ay/TaJcl mice were used in this study. The cognitive abilities and memory declined in the mice and worsened in the 50-week-olds. The levels of advanced glycation end products (AGEs), receptor for AGE (RAGE), and Iba1 in the hippocampus were increased in the mice compared to those in the control mice. Hippocampal levels of CC-chemokine receptor 7 and inducible nitric oxide synthase, which are from M1-type macrophages that shift from microglia, were higher in KK-Ay/TaJcl mice than in control mice. Tumor necrosis factor (TNF)-α and nitric oxide (NO) levels secreted by M1-type macrophages were similarly elevated in the mice and were even higher at the age of 50 weeks. NO levels were markedly elevated in the 50-week-old mice. In contrast, differentiation of CD163 and arginase-1 did not change in both mouse types. Memory and learning declined with age in diabetic mice, and the AGEs/RAGE/M1-type macrophage/NO and TNF-α pathways played an important role in exacerbating memory and learning in those mice.

## 1. Introduction

Aging is progressing in modern times, and the number of patients with lifestyle-related diseases is increasing. Lifestyle-related diseases are also involved in dementia and are deeply involved not only in vascular dementia (VD), but also in the development and progression of Alzheimer’s disease (AD). Among the lifestyle-related diseases occurring in middle-aged individuals, hypertension, diabetes, dyslipidemia, and metabolic syndrome increase the risk of developing AD and promote pathological degenerative processes [1].

Notably, diabetes increases the incidence of dementia in the elderly [2]. Elderly patients with diabetes have a higher incidence of VD than chronic vascular lesions, and recent epidemiological studies have reported that the risk of AD complications is also high [3,4,5]. The mechanisms by which diabetes modifies cognitive function can be divided into vascular and metabolic factors and could be reversible and irreversible [6]. AD-type dementia is related to metabolic factors and accumulates in the brain, causing neuropathy. Insulin-degrading enzymes break down amyloids and prevent their accumulation in the brain. When diabetes causes insulin resistance, a large amount of insulin is required (hyperinsulinemia); therefore, a large number of insulin-degrading enzymes are used. Consequently, brain amyloids cannot decompose and accumulate [7]. In addition, choline acetyltransferase activity is decreased in patients with AD, and cholinergic neuropathy is thought to occur, affecting memory and learning abilities [8]. Recent reports have shown that the receptor for advanced glycation end product (RAGE) is abundant in brain microglia and astrocytes of patients with AD, caused by inflammation and apoptosis, resulting from AGEs/RAGE [9,10,11]. However, the details of the onset mechanism of AD in the diabetic elderly are not known.

In this study, we used KK-Ay/TaJcl mice, a type 2 diabetes mouse model, for 50 weeks to investigate the mechanism of AD in elderly patients with diabetes. We then focused on microglia in the hippocampus of the mouse brain and examined the M1 and M2 shifts in macrophages.

## 2. Materials and Methods

### 2.1. Animal Experiments

Specific pathogen-free (SPF) 7- or 50-week-old C57BL/6N and KK-Ay/TaJcl male mice were obtained from CLEA Japan Inc. (Tokyo, Japan). The mice were separately kept in cages in specific pathogen-free conditions at 23 ± 1 °C, with a 12 h light:12 h darkness schedule. The mice were housed with unlimited access to drinking water and a pelleted basal diet. On the final day of the study, the body weights were measured. Blood glucose levels were measured from the tail vein of each mouse. We defined diabetic as more than a 300 mg/dL blood glucose level. In order to eliminate factors affecting the experiment, each animal was kept under constant conditions, such as free access to the same food, minimization of stress load, and freedom of movement within the cage. This work was conducted in accordance with the recommendations of the Guide for the Care and Use of Laboratory Animals of Suzuka University of Medical Science (approval number: 34). All dissections were performed under pentobarbital anesthesia. All efforts were made to minimize mouse suffering.

### 2.2. Open Field Test

The 50 × 50 × 40 cm^3^ open field area consisted of plastic. The mice’s distance moved (cm) was measured by a video tracking system (Smart2, Bio Research Center, Nagoya, Japan) during 15 min and was used as motor activity. The data obtained were graphed.

### 2.3. Step-Through Passive Avoidance Test

This test was used to evaluate non-spatial long-term memory following a previously described method [12]. The experimental apparatus (Bio Research Center, Nagoya, Aichi, Japan) consisted of two compartments: a light compartment and a dark compartment separated by a grid door. A stainless-steel shock grid floor was presented in the dark compartment. During the acquisition trial, the mouse was placed in the light compartment. After 60 s, the grid door between the compartments was opened. The step-through latency for animals to enter the dark compartment was measured, and the door was closed. As soon as the animals entered the dark compartment, there was an inescapable foot-shock (0.5 mA for 3 s). The retention test was performed 24 h after the training trial in a similar manner without the electric shock, and the step-through latency to the dark compartment was recorded. The maximal cutoff time for step-through latency was 600 s.

### 2.4. Measurement of AGEs, TNF-α, RAGE, Iba1, CCR7, iNOS, NO, CD163, and Arginase-1 in the Hippocampus

At the end of the study, the brains were harvested. Following this, the hippocampus was rapidly dissected, excised, and homogenized in lysis buffer (Kurabo, Osaka, Japan), and the lysate was centrifuged at 10,000 rpm. ELISA kits were used to measure the hippocampus concentrations of AGEs, tumor necrosis factor (TNF)-α, RAGE (MyBioSource, San Diego, CA, USA), cluster of differentiation (CD) 163 (Elabscience, Houston, TX, USA), ionized calcium binding adapter molecule 1 (Iba1: South San Francisco, CA, USA), CC-chemokine receptor 7 (CCR7: abbexa, Cambridge, UK), inducible nitric oxide synthase (iNOS), and arginase-1 (CUSABIO, Houston, TX, USA). NO was assayed using assay kits (NO_3_^−^+NO_2_^−^ colorimetric assay; Dojindo, Kumamoto, Japan) according to the manufacturer’s instructions. The microplate reader (Molecular Devices, Sunnyvale, CA, USA) was used for the measurement of optical density.

### 2.5. Statistical Analysis

The results are expressed as the means ± the standard deviation, and the data were analyzed using one-way ANOVA followed by Tukey’s post hoc test or the Steel–Dwass test was applied. The statistical significance level was set at *p* < 0.05 and 0.01.

## 3. Results

### 3.1. Effect of Aging on Body Weight and Blood Glucose Level in KK-Ay/Tajcl Mice

**Figure 1:**
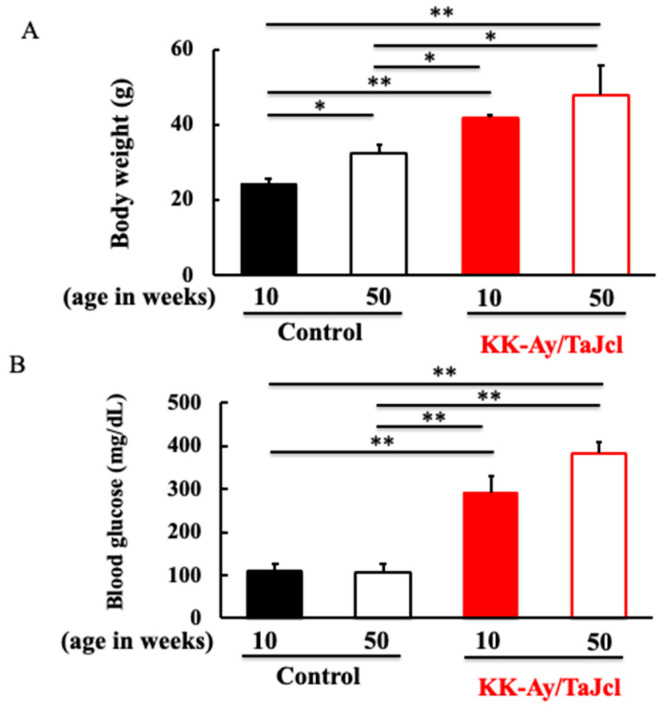
Effect of aging on body weight (**A**) and blood glucose levels (**B**) in KK-Ay/TaJcl mice. Values are expressed as the mean ± SD of five animals. * *p* < 0.05, ** *p* < 0.01. Control: C57BL/6j mice.

The body weight was higher in KK-Ay/Tajcl mice than in the controls. The body weight of the control mice increased with age, but that of the KK-Ay/Tajcl mice did not differ between 10 and 50 weeks of age (Figure 1A). The blood glucose levels were higher in the KK-Ay/Tajcl mice than in the control mice. In the control and KK-Ay/Tajcl mice, the blood glucose levels did not change with age; however, in the KK-Ay/Tajcl mice, the 50-week-old mice showed higher blood glucose levels than the 10-week-old mice (Figure 1B).

### 3.2. Behavioral Effects on Aging in KK-Ay/TaJcl Mice

**Figure 2:**
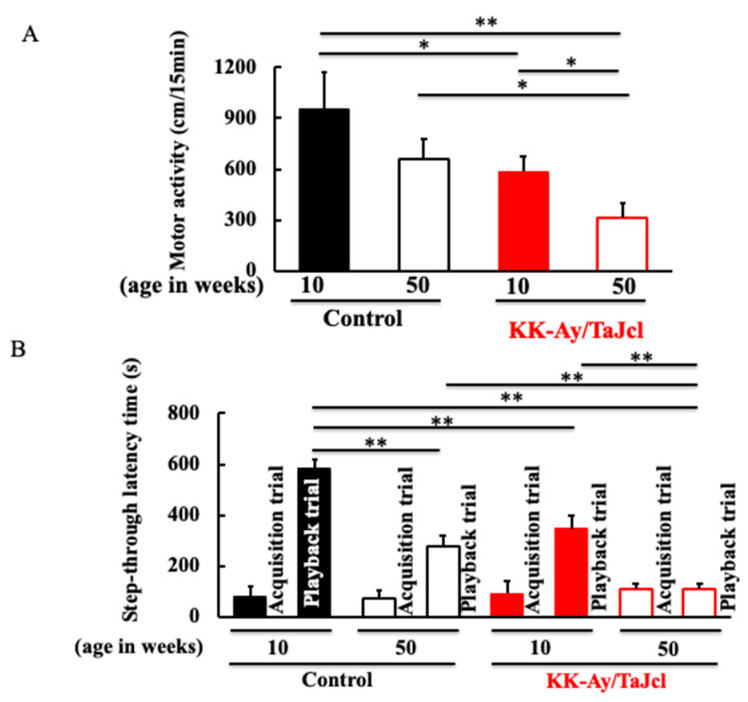
Effect of aging on motor activity (**A**) and memory and learning activity (**B**) in KK-Ay/TaJcl mice. The step-through passive avoidance test (**B**) was used to assess memory and learning abilities. Values are expressed as the mean ± the SD of five animals. * *p* < 0.05, ** *p* < 0.01. Control: C57BL/6j mice.

Locomotor activity was lower in the KK-Ay/Tajcl mice than in the controls. In both the control and KK-Ay/Tajcl mice, the 50-week-old mice showed decreased locomotion compared to the 10-week-old mice (Figure 2A). The control and KK-Ay/Tajcl mice had the same results in the first trial (conditioning: acquisition trial). In the second trial (playback trial), high values were obtained from animals with established memory and learning, and lower values indicate lower memory and learning ability. In both the control and KK-Ay/Tajcl mice, there were lower memory and learning abilities in the 50-week-old mice than in the 10-week-old mice. Memory and learning abilities were not observed in the 50-week-old KK-Ay/Tajcl mice (Figure 2B).

### 3.3. Effect of Aging on AGEs and RAGE in KK-Ay/TaJcl Mice

**Figure 3:**
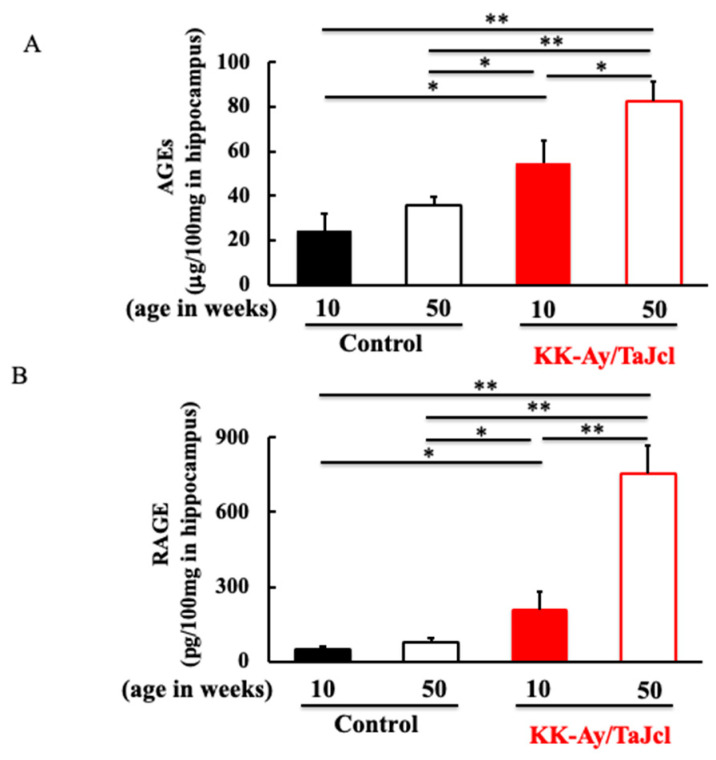
Effect of aging on levels of AGEs (**A**) and RAGE (**B**) in hippocampus of KK-Ay/TaJcl mice. The data show one representative experiment containing five animals. The hippocampus levels of AGEs and RAGE in mice were measured using ELISA. Values are expressed as the mean ± the SD derived from five animals. * *p* < 0.05, ** *p* < 0.01. Control: C57BL/6j mice.

AGEs are produced in large quantities in patients with diabetes. We investigated the expression levels of AGEs and their receptor, RAGE, which are involved in memory and learning, in the hippocampus. Both the AGE and RAGE levels were higher in the KK-Ay/Tajcl mice than in the controls (Figure 3A,B). In addition, in the KK-Ay/Tajcl mice, it significantly increased at 50 weeks of age compared to that at 10 weeks of age (Figure 3A,B).

### 3.4. Effect of Aging on Iba1, CCR7, iNOS, TNF-α, and NO in KK-Ay/TaJcl Mice

**Figure 4:**
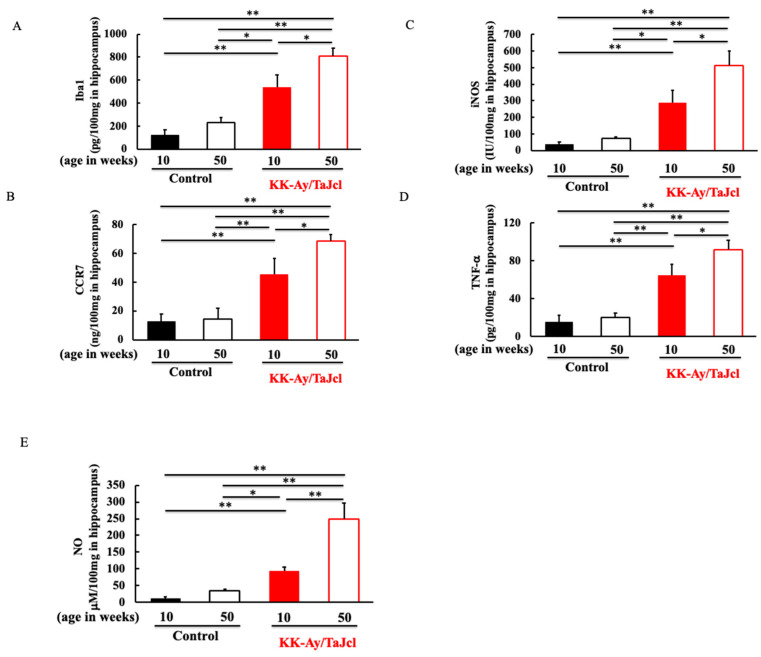
Effect of aging on the levels of Iba1 (**A**), CCR7 (**B**), iNOS (**C**), TNF-α (**D**), and NO (**E**) in the hippocampus of the KK-Ay/TaJcl mice. The data show one representative experiment containing five animals. The hippocampus levels of Iba1, CCR7, iNOS, and TNF-α in the mice were measured using an ELISA kit. The hippocampus level of NO was measured using the assay kit. Values are expressed as the mean ± the SD derived from five animals. * *p* < 0.05, ** *p* < 0.01. Control: C57BL/6j mice.

Next, we investigated the expression levels of microglia (Iba1) involved in AGEs, RAGE, and the M1/M2 switch in microglia. Firstly, we investigated the M1 macrophages [13]. The expression of Iba1 in the hippocampus was higher in the KK-Ay/Tajcl mice than in the control mice. Moreover, both the control and KK-Ay/Tajcl mice showed higher levels in the 50-week-old mice than in the 10-week-old mice (Figure 4A). CCR7 and iNOS, which are M1 macrophages, were higher in the KK-Ay/Tajcl mice than in the control mice, and their levels in the 50-week-old mice were higher than those in the 10-week-old mice (Figure 4B,C). In particular, the 50-week-old KK-Ay/Tajcl mice exhibited a remarkable increase. Furthermore, the TNF-α and NO secreted by the M1 macrophages were also increased in the KK-Ay/Tajcl mice at 50 weeks of age compared to those at 10 weeks of age (Figure 4D,E). In particular, NO production by iNOS was markedly increased in the 50-week-old KK-Ay/Tajcl mice (Figure 4E).

### 3.5. Effect of Aging on CD163 and Arginase-1 in KK-Ay/Tajcl Mice

**Figure 5:**
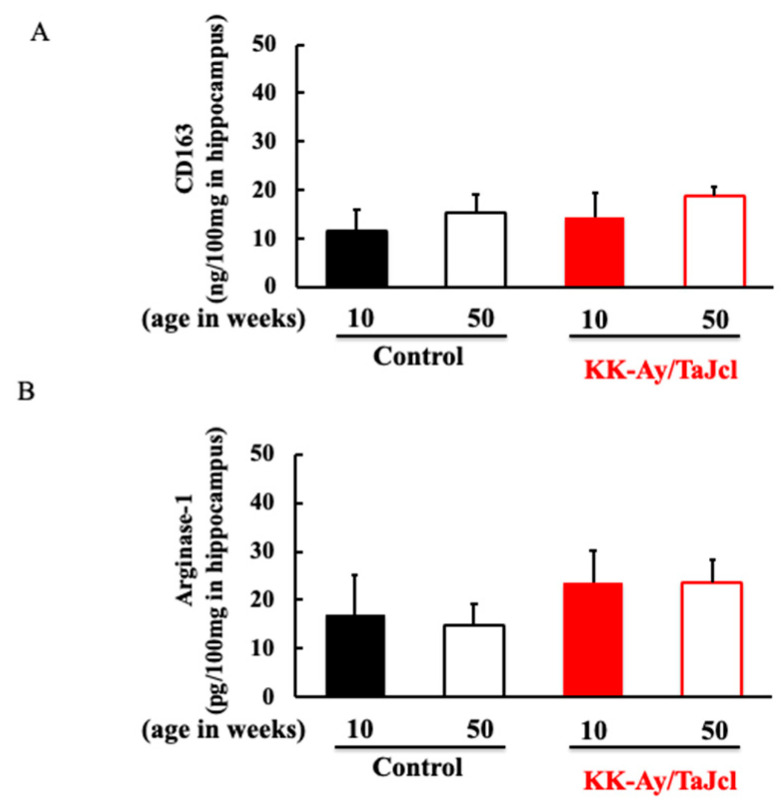
Effect of aging on levels of CD163 (**A**) and arginase-1 (**B**) in hippocampus of KK-Ay/TaJcl mice. The data show one representative experiment containing five animals. The hippocampus levels of CD163 and arginase-1 in the mice were measured using an ELISA kit. Values are expressed as the mean ± the SD derived from five animals. Control: C57BL/6j mice.

We confirmed the expression of M2 macrophages in the hippocampus, which antagonizes M1 macrophages. Neither CD163 nor arginase-1, the markers of M2 macrophages [13], differed between the groups (Figure 5A,B).

## 4. Discussion

With aging, many molecules that are recognized as foreign substances are produced [14]. Amyloids are well-known molecules that are found in the brain. In particular, amyloid-β, in which the APP protein has been cleaved, aggregates very easily, damages nerve cells, and activates microglia, which are macrophages in the brain [15]. Numerous senile plaques are found in the brains of patients with AD. Neuritic plaques are composed of amyloid-β, and in the presence of glucose, amyloid-β is AGE and aggregated [16]. Microglia and astrocytes in the brain contain RAGE, a receptor for AGEs, and the number of patients with AD is increasing compared to healthy elderly people [9,17]. In the present study, the expression levels of AGEs and RAGE in the hippocampus were increased in aged diabetic mice (Figure 3). Microglia are a kind of resident macrophage, and when activated, they secrete inflammatory cytokines such as TNF-α and IL-1β, which damage neurons [10,11].

Macrophages are functionally categorized into the M1 type, which acts injuriously, and the M2 type, which acts as an anti-inflammatory agent [18,19]. The M1/M2 classification has also been applied to brain-resident microglia [20]. In this study, hippocampal microglia shifted to the M1 type. IFN-g, LPS, and TNF-g induce the M1-type macrophages [20,21]. In diabetes, the production of TNF-α is increased [22]. We reported previously that aged diabetic mice secreted a large amount of TNF-α from liver and kidney [23]. AGEs, which are upregulated by hyperglycemia, bind to RAGE to increase TNF-α secretion [24]. This increased TNF-α induces M1-type microglia and increases the secretion of inflammatory cytokines, such as CCR7 and iNOS [20]. Furthermore, AGEs bind to RAGE expressed in microglia and increase the secretion of TNF-α from Iba1, thereby accelerating its shift to M1 [18]. However, when AGEs accumulate in the hippocampus and cause neuropathy, cathepsin B and E levels increase in microglia accumulated in the hippocampus prior to neuropathy. Cathepsin B binds to the transcription factor NF-kB and degrades the repressor IkB, which inactivates it and induces the gene conversion of damaging molecules. In addition, cathepsin E induces an increase in cathepsin B. Thus, it has been reported that these two processes transform macrophages into the neurotoxic M1 type [25]. In the present study, an increase in cathepsin E was observed in the hippocampus, suggesting that the inflammatory M1 type occupied the brains of the aged diabetic mice.

Notably, a marked increase in iNOS secretion was observed (Figure 4), which causes overproduction of NO and increases the release of inflammatory cytokines [26]. iNOS-derived NO is not only a major marker of endothelial function, but is also involved in cerebral circulation disorders [27]. Currently, cognitive and memory disorders, such as AD, are also involved in chronic oxygen supply reduction and metabolic disorders due to decreased cerebral circulation [28]. Cerebrovascular endothelial disorders are strongly associated with vascular risk factors (diabetes in this study) and aging [29,30]. These results suggest that a marked increase in iNOS in M1 macrophages impairs vascular endothelial function in aged diabetic mice and induces neuropathy due to the degeneration of the cerebral microvascular endothelium.

Unfortunately, our study has some weaknesses. First, in this test, the value was obtained using the ELISA kit. However, the source of each marker cannot be specified, and immunohistological analysis is required. Second, the use of FACS gives more specific results for the analysis of M1 and M2 macrophages. Thirdly, hippocampal findings can identify the tissue identity better by the immunohistological approach. In this way, it is necessary to investigate the immunohistology in more detail, and we will add further investigations.

**Figure 6:**
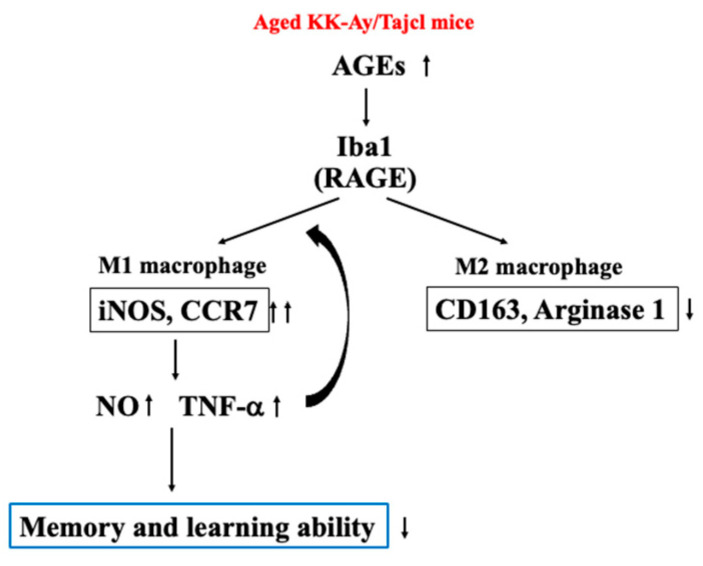
Mechanism of the effect of aging on memory and learning abilities in KK-Ay/TaJcl mice.

Thus, in aged diabetic mice, brain hippocampal AGEs activate microglia and shift to type 1 macrophages through the action of cathepsins and TNF-α. It has been suggested that M1-type macrophages secrete large amounts of inflammatory cytokines and NO, which cause neuropathy (Figure 6).

Many reports have been published on the factors that cause age-related type 2 diabetes, especially dementia and AD [31]. In AD, amyloid-β begins to accumulate in the brain, deposits in the brain as senile plaques, and kills nerve cells. Blood vessel pulsation and lipid metabolism are important for excreting this amyloid-β. However, in elderly people with diabetes mellitus, blood vessels are damaged, making pulsation less likely to occur, making it easier to induce dementia. In addition, the nerve energy of the brain is glucose, and brain nerve cells must always take in glucose, while insulin plays an important role. Glucose is taken up by glial cells, but in elderly diabetic patients, insulin that acts on glial cells is insufficient and glial cells are unable to take up glucose from the blood. Therefore, insulin resistance also influences the progression of dementia [32,33]. In this way, there are many factors between elderly type 2 diabetes and dementia, and it is necessary to examine the relationship with the results of this experiment.

## Data Availability

Not applicable.
